# Increased expression of the RNA-binding protein Musashi-2 is associated with immune infiltration and predicts better outcomes in ccRCC patients

**DOI:** 10.3389/fonc.2022.949705

**Published:** 2022-10-21

**Authors:** Hui Li, Xiaole Meng, Xuting You, Wenting Zhou, Wanxin Ouyang, Xin Pu, Runan Zhao, Huamei Tang

**Affiliations:** ^1^ Department of Pathology, Xiang’an Hospital of Xiamen University, Xiamen, China; ^2^ National Institute for Data Science in Health and Medicine, Xiamen University, Xiamen, China; ^3^ Organ Transplantation Institute of Xiamen University, Fujian Provincial Key Laboratory of Organ and Tissue Regeneration, School of Medicine, Xiamen University, Xiamen, China; ^4^ Department of Pathology, Changhai Hospital, Navy Medical University, Shanghai, China

**Keywords:** ccRCC, Musashi-2, immune, metabolism, prognosis

## Abstract

RNA-binding proteins (RBPs) mainly contribute to abnormalities in posttranscriptional gene regulation. The RBP Musashi-2, an evolutionarily conserved protein, has been characterized as an oncoprotein in various tumors. However, the prognostic value and potential roles of Musashi-2 in clear cell renal cell carcinoma (ccRCC) have not yet been elucidated. In this study, we found that Musashi-2 was mainly expressed in the normal distal tubular cells and collecting duct cells of the kidneys, while its expression was significantly decreased in ccRCC. And higher expression levels of Musashi-2 indicated better overall survival (*OS*) in ccRCC. Furthermore, immunohistochemistry demonstrated that PD-L1 expression was negatively correlated with Musashi-2 expression, and Musashi-2 was found to be remarkably correlated with multiple immune cells and immune inhibitors, including CD8^+^ T cells, CD4^+^ T cells, regulatory T (Treg) cells, PDCD1, CTLA4, Foxp3, and LAG3. Functional enrichment analysis revealed that Musashi-2 might be involved in ccRCC metabolic reprogramming and immune infiltration and further predicted the therapeutic sensitivity of ccRCC. Taken together, Musashi-2 is a prognostic biomarker for ccRCC patients that may provide novel insights into individualized treatment strategies and guide effective immunotherapy.

## Introduction

Clear cell renal cell carcinoma (ccRCC) is the most prevalent pathological subtype of kidney cancer (accounting for 70–80% of renal cell carcinoma cases) (1) and is characterized by inactivation of the von Hippel–Lindau (VHL) tumor suppressor gene and dysregulation of hypoxia-related signaling pathways (2). Although surgical resection is an effective treatment for early-stage ccRCC, 30% of patients still experience tumor recurrence or metastasis after surgery (3). Currently, the main reason for the poor prognosis of ccRCC patients is that distant metastases are present at the time of diagnosis, and the clinical effect of tyrosine kinase inhibitor (TKI) therapy is limited (4). Therefore, further discovery of more effective molecular biomarkers is crucial for achieving early diagnosis of tumors and improving patients’ quality of life.

RNA-binding proteins (RBPs) are a class of highly evolutionarily conserved proteins consisting of more than 2,000 members that interact with transcripts produced in various RNA-driven processes (5, 6). RBPs have been proven to maintain the physiological balance involved in the regulation of essential cellular processes, such as the splicing, modification, transport, localization, stability, degradation, translation and metabolism of RNA (7, 8). Previous studies have shown that some RBPs significantly contribute to the remodeling of the ccRCC transcriptome by regulating target mRNA stability (9–11). Recent studies have also indicated that the abnormal expression of some RBPs is involved in the occurrence, proliferation, metastasis, and progression of ccRCC (10–13). Furthermore, prognostic models related to RBPs have also been investigated with database data, which may provide a way to predict the prognosis of ccRCC (14–18). However, the precise roles of RBPs in ccRCC tumorigenesis and development remain unknown.

The RBP Musashi-2, a member of the Musashi gene family, has been characterized as an oncoprotein in a variety of tumors, and its aberrant expression is closely associated with tumor occurrence and progression (including in breast, lung, pancreatic, liver, prostate and colorectal cancers) (19–24) mediated by its participation in the regulation of various molecules and signaling pathways (25). Although some studies that comprehensively analyzed RBPs have shown that multiple prediction models based on RBPs have important guiding significance for the outcome of ccRCC, it is worth noting that these studies were based on increased expression levels of RBPs (15–18). However, the role and function of Musashi-2 in RCC, particularly in ccRCC, are still not clear, and there are currently no studies on the effects of the RBP Musashi-2 on ccRCC patients.

This study is the first to demonstrate that the role of Musashi-2 in ccRCC differs from that in other tumors owing to the pivotal functions of Musashi-2 in metabolic reprogramming and immune infiltration. Our results may shed light on the clinical importance of Musashi-2 in ccRCC and provide a possible predictive indicator for evaluating the prognosis of patients with ccRCC.

## Materials and methods

### Tissue microarrays 

A tissue microarray was purchased from Shanghai OUTDO Biotech Co., Ltd. (HkidE180Su02). It contained 144 cases of ccRCC tumor tissues and 29 cases of normal adjacent tissues available for statistical analysis after exclusion of tissue defects. The operation time range covered February 2008 to March 2010, the follow-up time ended in August 2015, and the follow-up period ranged from 5.5 to 7.5 years. Clinicopathological characteristics, including age, sex, tumor size, tumor grade, T classification, N classification, and M classification, were retrospectively collected. The T, N and M stages were determined according to the 7th edition of the American Joint Committee on Cancer (AJCC) TNM staging system.

### Hematoxylin and eosin staining

For histological analysis, sections were stained with H&E. Briefly, sections were incubated with a hematoxylin solution for 10 min to stain nuclei, and then they were incubated with an eosin solution for 3 min and transferred into an alcohol gradient (70% ethanol for 1 min, 90% ethanol for 1 min, and 100% ethanol for 1 min). Finally, the sections were dehydrated and cleared in xylene.

### Immunohistochemistry

Immunohistochemical staining was performed as described previously (26). Briefly, IHC was performed on the paraffin-embedded ccRCC tissues using Musashi-2 (Abcam, #ab76148), PD-L1 (GeneTech, #GT228002, Colone: ZR3), HIF1A (CST, (E1V6A), #48085), CA9 (MXB Biotechnologies, RAB-0615), KIT (MXB Biotechnologies, KIT-0029, Colone: YR145) and MKI67 (MXB Biotechnologies, MAB-0672, Colone: MX006) antibodies. IHC images were captured with a Leica Aperio Versa 200 microscope. Musashi-2 expression levels were scored by multiplying the percentage of positive cells by the staining intensity. The percentage was scored as follows: no positive cells, 0 point; 1%‐25%, 1 point; 26%‐50%, 2 points; 51%‐75%, 3 points; and 76%‐100%, 4 points. The staining intensity was scored as follows: no positive staining, 0 point; weak staining, 1 point; moderate staining, 2 points; and strong staining, 3 points. The final Musashi-2 IHC scores were classified as follows: 0‐4, low expression; and 6-12, high expression. For PD-L1 IHC evaluation, positive expression was defined as a tumor proportion score exceeding 1% (27).

### Analysis of the cancer genome atlas datasets using UALCAN

The ccRCC data in the TCGA database were analyzed using the UALCAN platform (http://ualcan.path.uab.edu). UALCAN is an online platform that contains raw TCGA data, including gene expression and clinicopathological data (28). In this study, the UALCAN database was employed to analyze the mRNA and protein expression of Musashi-2 in primary ccRCC tissues and its relationships with stage and grade.

### Survival analysis using TCGA datasets and the UCSC Xena platform

The prognostic value of Musashi-2 in ccRCC was evaluated using the TCGA datasets and the UCSC Xena Functional Genomics Explorer platform (https://xenabrowser.net/) (29). The Xena platform comprises gene expression profiles and survival information for multiple cancers, including survival and mRNA expression data for 607 ccRCC patients. The optimal cutoff value for survival was determined by the algorithms. In addition, samples lacking cancer results were excluded from the corresponding analyses.

### Analysis of Musashi-2 mRNA at the single-cell level and transcript level

The single-cell mRNA levels of Musashi-2 in the kidneys of normal humans were analyzed in data from The Human Protein Atlas (https://www.proteinatlas.org/). The mRNA expression in the cell type clusters identified in this study was visualized by a UMAP plot and a bar chart. Multiple transcripts of Musashi-2 were analyzed in normal kidney (GTEX) and TCGA ccRCC tissues by using the UCSC Xena platform (29).

### Immune infiltrate correlation analysis using TIMER and the Xena platform

Associations between immune cell infiltration and Musashi-2 expression were analyzed with the Tumor Immune Estimation Resource (TIMER) tool (https://cistrome.shinyapps.io/timer/). TIMER is an online tool for systematic analysis of immune infiltration in various cancers (30). In this study, the purity-corrected partial Spearman’s correlation (partial-cor) and p value provided by TIMER are shown in scatterplots, and immune infiltration estimations were calculated with immunedeconv-EPIC and CIBERSORT (31, 32). The correlations between immune inhibitors and Musashi-2 expression determined with the Xena platform are shown in heatmaps and scatterplots, and samples lacking results were excluded from the corresponding analysis.

### Functional enrichment analyses of Musashi-2

The limma package in R software was used to study the differentially expressed mRNAs in the Musashi-2 High (n=265) and Low (n=265) groups from the TCGA ccRCC datasets. “Adjusted P < 0.05 and Fold Change >1.5 or Fold Change < −1.5” were defined as the threshold for differential mRNA expression. The Cluster Profiler package (version: 3.18.0) in R was employed to analyze the Gene Ontology (GO)-identified functions of potential targets and perform Kyoto Encyclopedia of Genes and Genomes (KEGG) pathway enrichment analysis (33). The genes (top 100) similar to Musashi-2 were obtained using the Gene Expression Profiling Interactive Analysis platform (GEPIA2, http://gepia.cancer-pku.cn/) (34). Similar gene visualization of the functional enrichment analysis results was achieved by using an online bioinformatic platform from China (http://www.bioinformatics.com.cn/).

### Signaling pathways, drug half-maximal inhibitory concentration (IC50) values and tumor immune dysfunction and exclusion scores

To calculate signaling pathway scores, ccRCC RNA-sequencing (RNA-seq; FPKM) data were downloaded from the TCGA. The R software GSVA package (35) was used to perform single-sample gene set enrichment analysis (ssGSEA), and the correlations between Musashi-2 and pathway scores were analyzed by Spearman correlation analysis. For analysis of drug IC50 values, we predicted the therapeutic response for each sample based on the largest publicly available pharmacogenomic database [the Genomics of Drug Sensitivity in Cancer (GDSC), https://www.cancerrxgene.org/]. The prediction process involved the use of the R package “pRRophetic” (31), in which sample IC50 values were estimated by ridge regression and the prediction accuracy. All parameters were set to the default values with removal of the batch effect of combat and tissue type of all solid tumors, and duplicate gene expression data are summarized as the mean value. The potential therapeutic response to immune checkpoint blockade (ICB) was predicted with the TIDE algorithm as described previously (36). All the above analysis methods and R packages used the R foundation for statistical computing (2020) version 4.0.3.

### Statistical analysis

Clinical data analysis was conducted using GraphPad Prism 7.5 software. Differences between two groups were compared by using the Mann–Whitney test. Correlations were determined using Pearson correlation coefficient tests. Survival curve were plotted by the log-rank test with the hazard ratio (HR) and 95% confidence interval. Chi-squared tests were applied to analyze the relationships between Musashi-2 expression and clinicopathological features. A *p* value <0.05 was considered to indicate a significant difference.

## Results

### Increased Musashi-2 expression indicates good clinical outcomes in ccRCC

**Figure 1:**
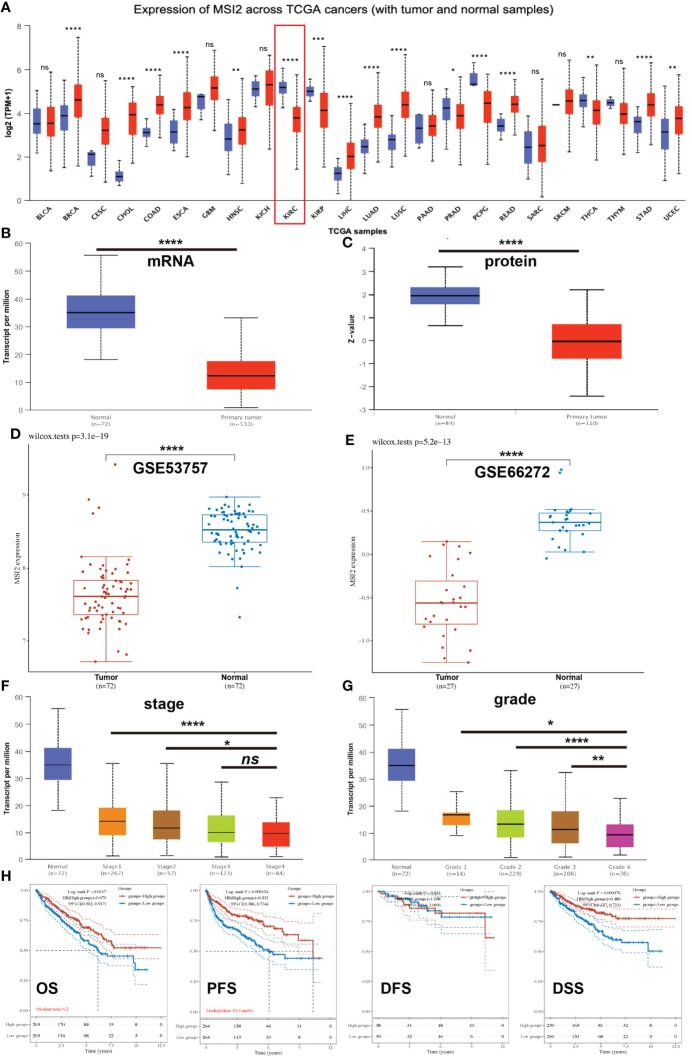
The expression of Musashi-2 and survival of ccRCC patients in the TCGA cohort. **(A)**, The expression of Musashi-2 in normal (N) and primary tumor (T) in the pancancer view. Blue, normal; and red, primary tumor. Sample size: BLCA: N=19, T=408; BRCA: N=114, T=1097; CESC: N=3, T=305; CHOL: N=9, T=36; COAD: N=41, T=286; ESCA: N=11, T=184; GBM: N=5, T=156; HNSC: N=44, T=520; KICH: N=25, T=67; KIRC: N=72, T=533; KIRP: N=32, T=290; LIHC: N=50, T=371; LUAD: N=59, T=515; LUSC: N=52, T=503; PAAD: N=4, T=178; PRAD: N=52, T=497; PCPG: N=3, T=179; READ: N=10, T=166; SARC: N=2, T=260; SKCM: N=1, T=472; THCA: N=59, T=505; THYM: N=2, T=120; STAD: N=34, T=415; UCEC:N=35, T=546. **(B, C)**, The mRNA and protein expression of Musashi-2 in normal n=72; n=84 and primary ccRCC n=533; n=110 tissues. **(D, E)**, The mRNA expression of Musashi-2 in the GSE53757 (Tumor=72, Normal=72) and GSE66272 (Tumor=27, Normal=27) datasets, statistical analyses were performed by using the Wilcox test. **(F, G)**, The mRNA expression of Musashi-2 related to the clinical stage and grade of ccRCC patients. **(H)**, Overall survival (*OS*), progression-free survival (*PFS*), disease-free survival (*DFS*) and disease-specific survival (*DSS*) of ccRCC patients based on Musashi-2 expression. The cutoff value was set at 50%, and survival was analyzed by the log-rank test. ns, not significant; *p < 0.05; **p < 0.01; ***p < 0.001; ****p < 0.0001.

As an oncogene, Musashi-2 has been found to exhibit increased expression in a variety of tumors. However, there are currently no studies on Musashi-2 in ccRCC. Therefore, we first analyzed the expression of Musashi-2 across cancers, including colon adenocarcinoma (COAD), breast cancer (BRCA), esophageal cancer (ESCA), and lung adenocarcinoma (LUAD), etc (**Figure 1A**). Unlike the findings for other types of tumors, the mRNA and protein expression levels of Musashi-2 in normal kidney tissues were significantly higher than those in cancer tissues (**Figures 1B, C**). This finding was further confirmed with the GSE53757 and GSE66272 datasets (37, 38)(**Figures 1D, E**). The associations of Musashi-2 mRNA expression with the stage and grade of ccRCC were also analyzed. As illustrated in **Figures 1F, G**, Musashi-2 mRNA expression was closely associated with the cancer stage and grade of ccRCC patients. We further assessed the prognostic value of Musashi-2 mRNA expression in patients with ccRCC. Surprisingly, lower mRNA expression levels of Musashi-2 were significantly related to poorer overall survival (*OS*), progression-free survival (*PFS*) and disease-specific survival (*DSS*) in patients with ccRCC (**Figures 1H**, **S1A, B**). Collectively, these results indicate that Musashi-2 is significantly downregulated in ccRCC and the lower expression predicts a poorer prognosis in ccRCC patients.

### Abnormal features of Musashi-2 in normal kidney and ccRCC tissues

**Figure 2:**
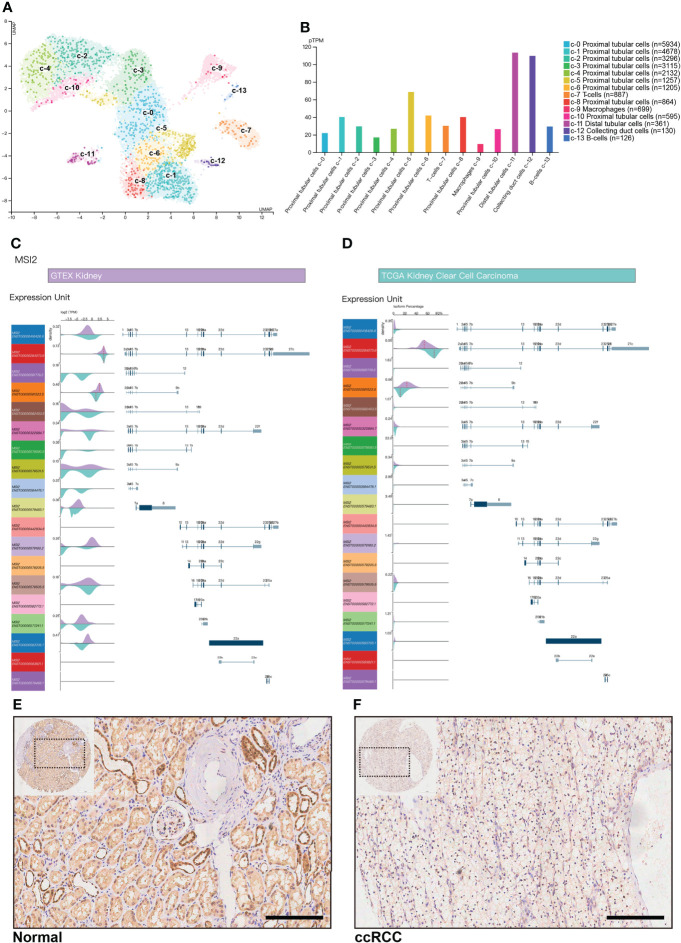
Musashi-2 mRNA transcripts and expression in normal kidney and ccRCC tissues. **(A, B)**, Musashi-2 mRNA expression in single cell type clusters identified in the normal kidneys visualized in a UMAP plot **(A)** and a bar chart **(B)**, including 9 proximal tubular cell clusters, 1 distal tubular cell cluster, 1 collecting duct cell cluster, and 3 immune cell clusters. **(C, D)**, Musashi-2 transcript expression unit (TPM) **(C)** and isoform percentage **(D)** in GTEX normal kidney and TCGA ccRCC data, 19 transcripts of Musashi-2 were analyzed, purple, normal kidney; and cyan, KIRC. **(E, F)**, Representative image of immunohistochemical staining for Musashi-2 in normal **(E)** and ccRCC **(F)** tissues. Scale bars, 200 μm.

To explore why abnormal expression of Musashi-2 appears in the kidneys, we obtained a single-cell map of the kidneys from The Human Protein Atlas. Normal kidney tissue cells were divided into 14 single-cell subpopulations, including 9 proximal tubular cell clusters, 1 distal tubular cell cluster, 1 collecting duct cell cluster, and 3 immune cell clusters. The mRNA expression levels of Musashi-2 in the distal tubular cell cluster-c11, collecting duct cell cluster-c12 and proximal tubular cell cluster-c5 were higher than those in the other cell clusters (**Figures 2A, B**). We further analyzed all transcripts of Musashi-2 in normal kidney and ccRCC tissues. It was found that 14 of the 19 transcripts of Musashi-2 were mainly expressed in kidney, with the transcripts ENST00000284073.6 and ENST00000581523.5 accounting for a relatively large proportion (total proportion exceeded 80%) (**Figures 2C, D**). In addition, some transcripts including ENST00000581776.5, ENST00000579590.5 and ENST00000584476.1were only expressed in ccRCC; but they were scarcely expressed in the normal kidneys. Most importantly, the expression abundance (TPM) of the transcript ENST00000581523.5, accounting for a large proportion in the isoform percentage, was significantly reduced in ccRCC, whereas the expression abundance (TPM) of the transcripts ENST00000284073.6 was similar in the normal kidney and ccRCC (**Figures 2C, D**). We further assessed the expression of Musashi-2 by immunohistochemical staining of a TMA including 29 normal kidney tissues and 144 primary ccRCC tissues. Our immunohistochemical results were consistent with the above finding that the expression of Musashi-2 in normal kidney tissues was mainly distributed in the distal renal tubules and collecting ducts and was higher than that in cancer tissues (**Figures 2E, F**). In conclusion, the expression characteristics of Musashi-2 in normal kidney and ccRCC tissues are abnormal.

### Musashi-2 is negatively correlated with PD-L1 expression in ccRCC

**Figure 3:**
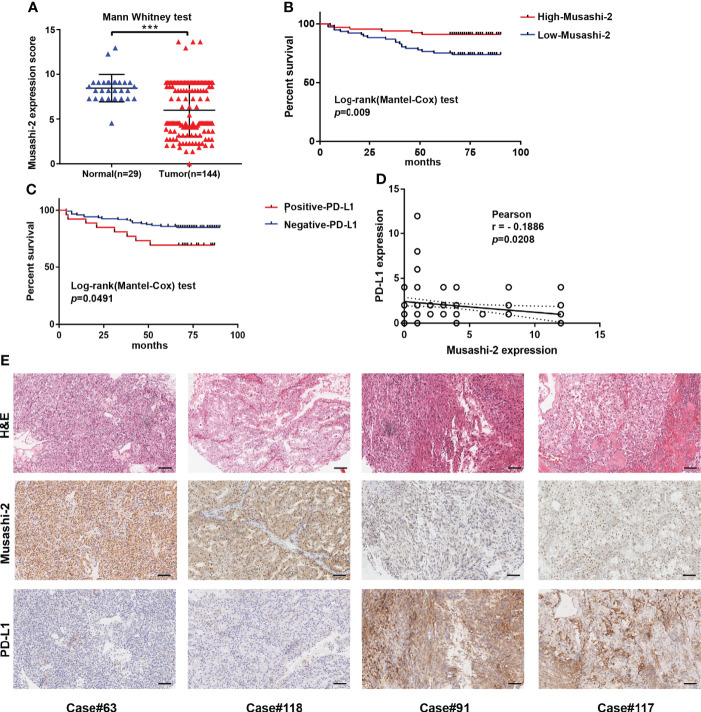
Musashi-2 and PD-L1 expression in 144 clinical ccRCC and 29 adjacent specimens. **(A)**, Musashi-2 expression in 144 clinical ccRCC tissues and 29 adjacent tissues determined by IHC. Statistical analyses were performed by using the Mann–Whitney test. **(B, C)**, Survival analysis based on Musashi-2 **(B)** and PD-L1 **(C)** expression in clinical ccRCC. Statistical analyses were performed by using the log-rank (Mantel–Cox) test. **(D)**, Associations between Musashi-2 and PD-L1 expression measured by IHC determined by using the Pearson correlation coefficient test. **(E)**, Representative image of H&E staining and immunohistochemical staining for Musashi-2 and PD-L1 in ccRCC tissues. Scale bars, 100 μm. ***p < 0.001.

To explore the potential effects of Musashi-2 in ccRCC, we first analyzed the significance of Musashi-2 in our clinical TMA. Musashi-2 expression was significantly downregulated in tumor tissues compared with adjacent tissues (**Figure 3A**, **Table 1**). Furthermore, as shown in **Table 2**, Musashi-2 expression was closely associated with tumor grade (*p=0*.006) in ccRCC patients, while the associations with other clinical features were not significant. In our cohort of 144 cases, we found that the patients with higher expression of Musashi-2 also had a better prognosis (*p*=0.009) (**Figure 3B**). The results of univariate and multivariate Cox analyses indicated that Musashi-2 expression could act as an independent prognostic factor for ccRCC (**Table 3**). Moreover, we performed immunohistochemical staining analysis of PD-L1 in the TMA. However, the patients with higher expression of PD-L1 had a poorer prognosis (*p*=0.0491) (**Figure 3C**). Notably, the patients with higher Musashi-2 expression had lower PD-L1 expression, and Musashi-2 expression was slightly negatively correlated with PD-L1 expression (*p*=0.0208, r=-0.1886) (**Figures 3D, E**). In conclusion, the expression of Musashi-2 in ccRCC is associated with clinicopathological characteristics and PD-L1, and can act as an independent prognostic factor for ccRCC.

**Table 1:** Differential expression of Musashi-2 in 144 ccRCC tumors and 29 adjacent normal tissues.

	n	Musashi-2 expression		Chi-square value	*p* value
		High	Low		
Tumor	144	67	77	26.803	<0.001
Adjacent	29	28	1

**Table 2:** Correlation between Musashi-2 expression and clinicopathological characteristics in 144 ccRCC tumors.

	Variables	Musashi-2 expression		Total	χ^2^	*p* value
		Low	High		
Age (year)					2.606	0.106
	≤60	44	47	91		
	>60	33	20	53		
Sex					1.171	0.279
	male	58	45	103		
	Female	19	22	41		
Grade					7.454	0.006**
	I/II	61	39	100		
	III/IV	16	28	44		
T stage					1.818	0.178
	T1a	35	38	73		
	T1b-T3	42	29	71		
N stage					0.499	0.480
	N0	76	65	141		
	N1/N2	1	2	3		
TNM stage					0.140	0.709
	I	65	55	120		
	II-IV	12	12	24		
Tumor size					0.001	0.996
	≤3.5cm	31	27	58		
	>3.5cm	46	40	86		

**p < 0.01.

**Table 3:** Univariate and multivariate analyses of the factors correlated with Overall survival of 144 ccRCC patients.

Variables	Univariate analysis				Multivariate analysis			
	*p* value	HR	95%CI		*p*value	HR	95%CI	
expression	0.014	0.317	0.127	0.789	0.002	0.221	0.087	0.567
Age	0.053	2.140	0.990	4.627				
sex	0.119	0.428	0.148	1.243				
Tumor size	0.050	2.488	0.999	6.197				
Grade stage	<0.001	4.497	2.039	9.919	0.008	3.612	1.404	9.291
TNM stage	<0.001	8.621	3.965	18.746	0.014	3.907	1.313	11.620
T stage	0.003	3.946	1.584	9.831	0.533	1.435	0.461	4.464
N stage	<0.001	25.416	6.651	97.125	0.045	4.147	1.031	16.671

### Tumor-infiltrating immune cells are associated with Musashi-2 in ccRCC

**Figure 4:**
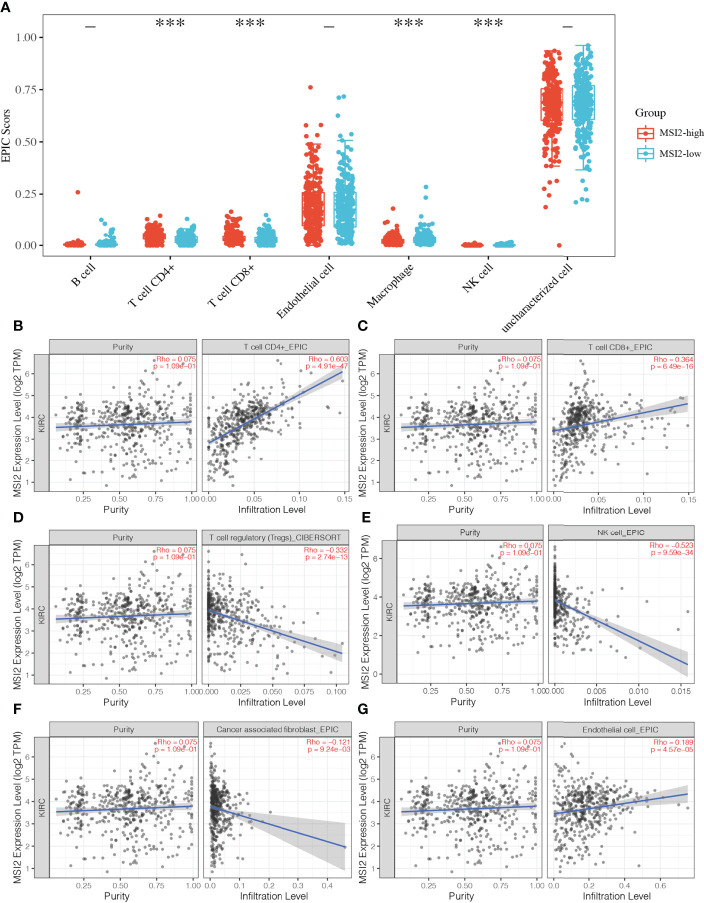
Associations between Musashi-2 and tumor-infiltrating immune cells in ccRCC. **(A)**, The distribution of immune EPIC scores in ccRCC patients with high (n=265) or low (n=265) Musashi-2 expression. Immune infiltration estimations were performed with immunedeconv-EPIC, the abscissa represents immune cell types, and the ordinate represents the expression distribution of immune score in different groups, blue, Musashi-2 low patients; and red, Musashi-2 high patients, statistical analyses were performed by using the Wilcox test. **(B–G)**, Associations between Musashi-2 and tumor-infiltrating immune cells including CD4^+^ T cells **(B)**, CD8^+^ T cells **(C)**, Treg cells **(D)**, NK cells **(E)**, cancer-associated fibroblasts **(F)** and endothelial cells **(G)** in ccRCC, (n=533). The left panels show tumor purity. ***p < 0.001.

In view of the fact that Musashi-2 expression predicted a better prognosis and was negatively correlated with PD-L1 expression, we speculated whether Musashi-2 affected the tumor immune microenvironment. We first compared the immune infiltration in TCGA ccRCC patients with high and low expression of Musashi-2 and found that CD4^+^ and CD8^+^ T cells were significantly increased in the high Musashi-2 expression group (**Figure 4A**). Then, we further analyzed the relationships between Musashi-2 expression and the infiltration of various types of cells in the tumor microenvironment. The results showed that the expression level of Musashi-2 was significantly positively correlated with the infiltration of CD8^+^ T cells (Rho=0.364) and CD4^+^ T cells (Rho=0.603) (**Figures 4B, C**) but negatively correlated with that of regulatory T (Treg) cells (Rho=-0.332) and natural killer (NK) cells (Rho=-0.523) (**Figures 4D, E**). Cancer-associated fibroblast (Rho=-0.121) and endothelial cell (Rho=0.189) infiltration was also evaluated (**Figures 4F, G**). Collectively, these results indicate that Musashi-2 expression is closely associated with immune infiltration, especially CD4^+^ T-cell, CD8^+^ T-cell and Treg-cell infiltration.

### Musashi-2 expression is inversely associated with immune inhibitors in ccRCC

**Figure 5:**
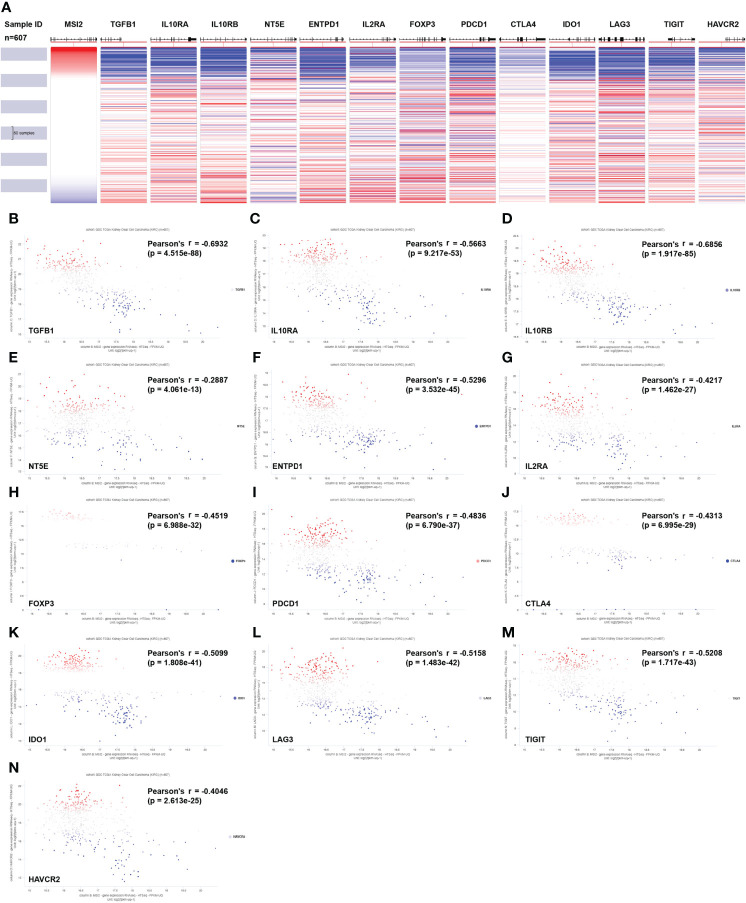
Associations between Musashi-2 and immune inhibitors in 607 cases of ccRCC. **(A)** Heatmap of Musashi-2 and immune inhibitor genes expression in ccRCC (n=607). **(B–N)**, Associations between Musashi-2 and immune inhibitors including TGFB1 **(B)**, IL10RA **(C)**, IL10RB **(D)**, NT5E **(E)**, ENTPD1 **(F)**, IL2RA **(G)**, FOXP3 **(H)**, PDCD1 **(I)**, CTLA4 **(J)**, IDO1 **(K)**, LAG3 **(L)**, TIGIT **(M)** and HAVCR2 **(N)** in ccRCC (n=607) were shown in Scatter plots, blue plots represent patients with low expression of the indicated genes, red plots represent patients with high expression of the indicated genes, and grey or white plots indicate patients with no significance of the indicated genes.

Treg cells play an important inhibitory role in antitumor immunity, and Muashi-2 expression was inversely correlated with PD-L1 expression and Treg-cell infiltration in ccRCC (**Figures 3D**, **4D**). To explore how Musashi-2 affects tumor immune infiltration, we focused on the profiles of key immune inhibitors in ccRCC, and the relationships between Musashi-2 and immune inhibitors in 607 cases of ccRCC were evaluated. In cases with high expression of Musashi-2, the levels of inhibitory immunoregulators were significantly downregulated (**Figure 5A**). Further correlation analysis revealed that the expression of Musashi-2 was negatively correlated with the expression of TGFB1 (r=-0.6932), IL10RA (r=-0.5663), IL10RB (r=-0.6856), NT5E (r=-0.2887), ENTPD1 (r=-0.5296), IL2RA (r=-0.4217), FOXP3 (r=-0.4519), PDCD1 (r=-0.4836), CTLA4 (r=-0.4313), IDO1 (r=-0.5099), LAG3 (r=-0.5158), TIGIT (r=-0.5208), and HAVCR2 (r=-0.4046) (**Figures 5B–N**). Additionally, the roles of Musashi-2 and immune inhibitors in the prognosis of 602 ccRCC cases were also assessed, and high expression of Musashi-2 in ccRCC was associated with a better prognosis (*p*=0.02164), while high expression of immune inhibitors, including IL10RA (*p*=0.0723), IL10RB (*p*=0.01748), FOXP3 (*p*=0.00009978), PDCD1 (*p*=0.02439), CTLA4 (*p*=0.000272), LAG3 (*p*=0.001281) and TIGIT (*p*=0.01299), was associated with a poor prognosis (**Figures S2A–N**). In summary, these results indicate that Musashi-2 expression is negatively correlated with the expression of immune inhibitors.

### Functional enrichment analysis of Musashi-2 in ccRCC-metabolic reprogramming and immune regulation

Based on the above results, we inferred that Musashi-2 may regulate immune infiltration in ccRCC, but the biological function of this regulation remained unknown, leaving us to wonder whether it could affect signal transduction in related pathways. First, the associations between Musashi-2 expression and major signaling pathway scores were analyzed. The expression of Musashi-2 was negatively correlated with anti-inflammatory IL-10 (*p* =1.74e-10, cor =-0.27), the cellular response to hypoxia (*p* =2.22e-23, cor =-0.41), P53 signaling (*p* =7.75e-15, cor =-0.33), tumor proliferation (*p* =4.24e-17, cor =-0.35), angiogenesis (*p* =2.62e-12, cor =-0.30) and apoptosis (*p* =8.51e-11, cor =-0.28) (**Figures S3A–F**). Then, we further analyzed the association of Musashi-2 with metabolism-related eigengenes in ccRCC (**Figure S4A**). It was found that Musashi-2 expression was positively correlated with VHL (*p* =3.495e-23, r =0.3875), HIF1A (*p* =3.490e-24, r =0.3957) and KIT (CD117) (*p* =2.771e-58, r =0.5904) but negatively correlated with EPAS1 (*p* =6.674e-18, r =-0.3401), VEGFA (*p* =3.589e-57, r =-0.5857), SLC2A1 (*p* =8.422e-58, r =-0.5884), CA9 (*p* =4.302e-123, r =-0.7759), MKI67 (*p* =6.728e-35, r =-0.4714) and TP53 (*p* =3.270e-22, r =-0.3794) (**Figures S4B–J**). Among these genes, VHL, HIF1A, VEGFA, SLC2A1 and CA9 play critical functions in ccRCC development, tumorigenesis, energy metabolism and angiogenesis (2, 39). The immunohistochemical features of HIF1A, CA9, MKI67 and CD117 were further verified in our clinical specimens, and Musashi-2 expression was positively correlated with HIF1A, but negatively correlated with CA9 (**Figure S4K**).

**Figure 6:**
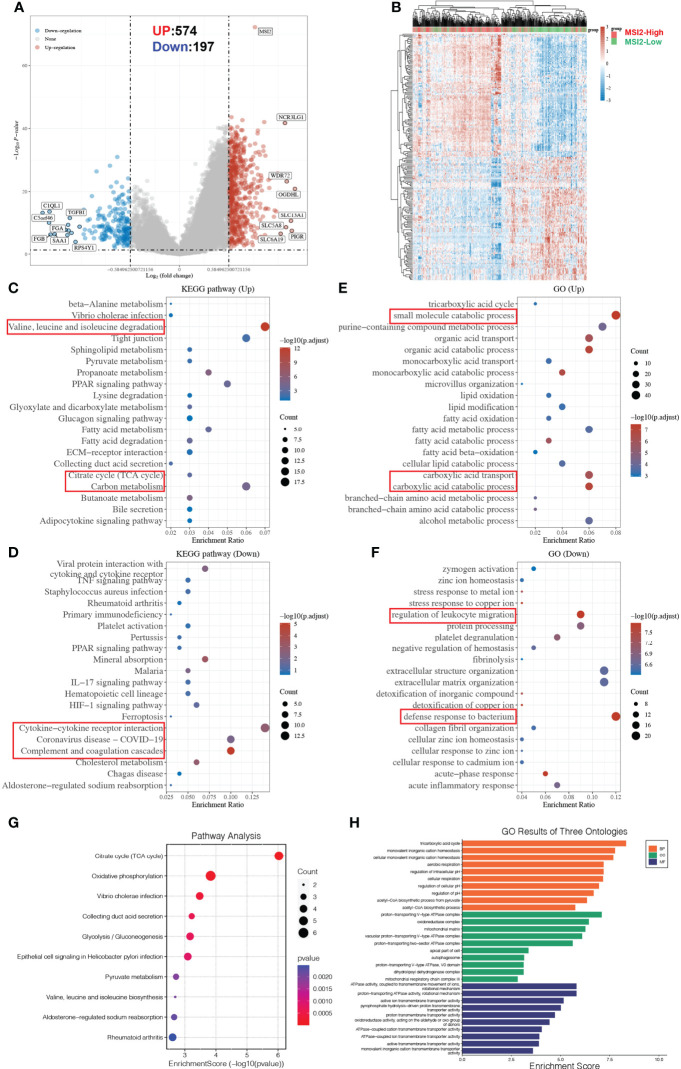
Metabolic reprogramming and functional enrichment analyses of Musashi-2-associated genes in ccRCC. **(A, B)**, Volcano plot **(A)** and heatmap **(B)** of the differential gene expression between ccRCC patients with high (n=265) or low (n=265) Musashi-2 expression from GDC TCGA, “Adjusted *p* < 0.05 and Fold Change >1.5 or Fold Change < −1.5” were defined as the threshold for differential mRNA expression. **(C, D)**, Functional enrichment of differentially expressed genes in up- and downregulated KEGG pathways, the up-regulated KEGG pathways include valine, leucine and isoleucine degradation, citrate cycle (TCA cycle) and carbon metabolism, the downregulated KEGG pathways include cytokine–cytokine receptor interaction, coronavirus disease-COVID-19 and complement and coagulation cascades. **(E, F)**, Functional enrichment of differentially expressed genes in up- and downregulated GO terms, the up-regulated GO terms include small molecule catabolic process, carboxylic acid transport and catabolic process, the downregulated GO terms include regulation of leukocyte migration and defense response to bacterium. **(G)**, Enrichment analysis of KEGG pathways based on the top 100 genes similar to Musashi-2, which are mainly enriched in the tricarboxylic acid cycle and oxidative phosphorylation. **(H)**, Enrichment analysis of GO terms based on the top 100 genes similar to Musashi-2, which are mainly enriched in the tricarboxylic acid cycle.

More importantly, the differentially expressed genes between the Musashi-2 high and low groups in the TCGA ccRCC datasets showed that there were 574 upregulated genes and 197 downregulated genes (**Figures 6A, B**). The differentially expressed genes were then analyzed by KEGG and GO enrichment analyses. The upregulated KEGG pathways and GO terms mainly included valine, leucine and isoleucine degradation; carbon metabolism; and small molecule catabolic process. In contrast, the downregulated KEGG pathways and GO terms mainly included cytokine–cytokine receptor interaction, coronavirus disease-COVID-19 and regulation of leukocyte migration (**Figures 6C–F**). Additionally, the top 100 genes similar to Musashi-2 were obtained with GEPIA2 (**Supplementary Table 1**). The biological functions of these genes were also explored by GO annotation and KEGG pathway analyses. As shown in **Figure 6G**, the top 10 KEGG pathways with *p* < 0.05 for the genes similar to Musashi-2 were determined. KEGG pathways such as hsa00020 (citrate cycle (TCA cycle)), hsa00190 (oxidative phosphorylation), hsa00010 (glycolysis/gluconeogenesis) and hsa00620 (pyruvate metabolism) were correlated with the functions of the genes similar to Musashi-2 in ccRCC. In addition, the GO annotation results are shown in **Figure 6H**. Biological process (BP) terms such as GO:0006099 (tricarboxylic acid cycle) and GO:0006086 (acetyl-CoA biosynthetic process from pyruvate), cellular component (CC) terms such as GO:0033176 (proton-transporting V-type ATPase complex) and GO:1990204 (oxidoreductase complex), and molecular factor (MF) terms such as GO:0044769 (ATPase activity, coupled to transmembrane movement of ions, rotational mechanism) and GO:0046961 (proton-transporting ATPase activity, rotational mechanism) were prominently related to the genes similar to Musashi-2. Taken together, these results indicate that Musashi-2 may regulate multiple signaling pathways, especially tumor metabolic processes and immune regulation in ccRCC.

### Targeted TKI drug IC50 and ICB TIDE score predictions based on Musashi-2 expression in ccRCC

**Figure 7:**
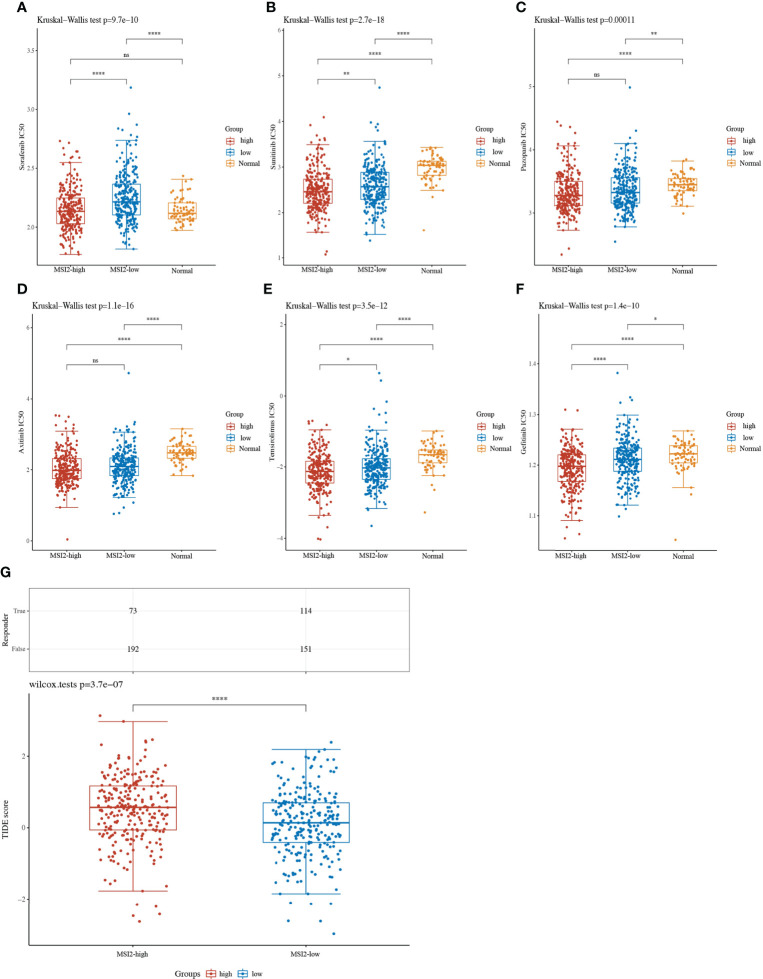
Targeted TKI drug IC50 values and ICB response prediction based on Musashi-2 in 530 cases of ccRCC. **(A–F)**, The distribution of IC50 values for targeted drugs including sorafenib **(A)**, sunitinib **(B)**, pazopanib **(C)**, axitinib **(D)**, temsirolimus **(E)** and gefitinib **(F)** in ccRCC tissues with high (n=265) or low (n=265) Musashi-2 expression and normal tissues (n=72) based on tumor RNA-seq data from GDC TCGA, statistical analyses were performed by using the Kruskal-Wallis test. **(G)**, The distribution of immune checkpoint blockade (ICB) response-TIDE scores in the Musashi-2 high (n=265) and low (n=265) ccRCC groups based on tumor RNA-seq data from GDC TCGA, statistical analyses were performed by using the Wilcox test. ns, not significant; *p < 0.05; **p < 0.01; ****p < 0.0001.

At present, the first-line targeted TKIs for advanced or metastatic renal cancer mainly include sorafenib, sunitinib, temsirolimus, gefitinib, pazopanib and axitinib. We wondered whether the expression level of Musashi-2 is related to the clinical response to targeted drug therapy. We further predicted the IC50 values for first-line treatments for ccRCC in the high and low Musashi-2 expression groups. It was found that in ccRCC patients with high Musashi-2 expression, sorafenib, sunitinib, temsirolimus and gefitinib had lower IC50 values (**Figures 7A, B, E, F**), reflecting higher sensitivity to targeted drug treatment. In contrast, pazopanib and axitinib did not show significant differences (**Figures 7C, D**). In addition, consistent with the above conclusion that Musashi-2 expression was negatively correlated with immunosuppressive factors, TIDE scores were used to predict the tumor response to ICB treatment, and patients with lower Musashi-2 expression were found to be more responsive to ICB treatment (**Figure 7G**). Based on the above findings, we concluded that the expression level of Musashi-2 may guide responsiveness to clinical therapeutic options, such as TKI or ICB treatment.

## Discussion

ccRCC is characterized by high mortality. Most patients are already in an advanced stage of disease at diagnosis, and there are currently no biomarkers for prognostic assessment, resulting in relatively poor outcomes. Therefore, the identification of sensitive biomarkers, a comprehensive understanding of potential functions, accurate prognostic assessment and the selection of appropriate treatments will facilitate improvements in the survival of ccRCC patients.

The relationships between the expression of various RBPs and the prognosis of ccRCC patients have attracted extensive attention due to recent studies suggesting that RBPs have important effects on ccRCC tumor initiation, progression, and metastasis and the immune microenvironment. Several RBP-related gene prognostic models have been identified as indicators for ccRCC (14–16, 18). To date, only Xing et al. identified a six-RBP gene model profile associated with immune infiltration (16), and no studies have attempted to investigate the precise role and function of the RBP Musashi-2 in ccRCC. In this study, we demonstrated that Musashi-2 is an independent prognostic factor for *OS* in ccRCC and that high Musashi-2 expression indicates good survival. Analyses of both database data and our clinical specimens confirmed this finding. Previous studies have shown that Musashi-2 is significantly upregulated in various tumors compared with the corresponding normal tissue, with these studies mainly focusing on the involvement of Musashi-2 in biological behaviors, such as cell stemness, proliferation and metastasis, as illustrated in **Figure 1A**. In contrast, we found for the first time that the expression level of Musashi-2 was downregulated in ccRCC, especially that of the transcript ENST00000581523.5. More importantly, the expression of Musashi-2 was negatively correlated with the immune inhibitor PD-L1 in our clinical specimens. PD-L1 plays a key role in maintaining the immunosuppressive tumor microenvironment, and the expression of tumor immunosuppressive factors determines the fate and survival status of patients (40). The involvement of Musashi-2 in the regulation of the immune microenvironment, such as modulation of allergic inflammation and interleukin secretion, has also recently been proven (22, 41).

In addition, the results of the immune infiltration analysis showed that Musashi-2 expression was positively correlated with CD4^+^ and CD8^+^ T cells but negatively correlated with Treg-cell infiltration. Based on the inverse correlations between Musashi-2 expression and Treg-cell infiltration or PD-L1 expression, further associations between immune inhibitors and Musashi-2 suggest that they also have opposite patterns in terms of ccRCC prognosis. Previous studies have shown that metabolic competition between tumor and immune cells may lead to tumor immunosuppression and progression (42). Energy metabolism also modulates immune activity in the tumor immune microenvironment, in which glycolysis can enhance immune activity (43). Morphologically, ccRCC cells are rich in glycogen, suggesting that aberrant glucose metabolism mainly changes during ccRCC development. To elucidate the potential underlying mechanism by which Musashi-2 regulates metabolic reprogramming and immune infiltration, we performed differential gene expression analysis to identify biological processes and signaling pathways enriched in the TCGA ccRCC cohort. Musashi-2 expression was found to be negatively correlated with the anti-inflammatory effect of IL-10, the cellular response to hypoxia, the p53 signaling pathway, tumor proliferation, angiogenesis and apoptosis. Notably, the biological functions of Musashi-2 and similar genes explored by GO annotation and KEGG pathway analyses revealed that Musashi-2 was upregulated in the tricarboxylic acid cycle and other carbon metabolism-related pathways but downregulated in cytokine–cytokine receptor interactions and the regulation of leukocyte migration. These findings suggest that Musashi-2 may regulate tumor immune invasion through ccRCC metabolic reprogramming. And recent studies have also shown that Musashi-2 is involved in metabolism related pathways to regulate cancer development and progression, including the reprogramming of branched-chain amino acids metabolism in myeloid leukaemia (44) and the regulation of glycolipid metabolism in Glioma (45).

Moreover, cancer immunotherapy has exhibited effectiveness in the treatment of various cancers. ICB treatments, such as targeting CTLA-4, PD-1 or PD-L1, are being clinically used for the treatment of diverse cancers. Nevertheless, many cancer patients exhibit a limited response or no response to immune checkpoint inhibitors (46, 47). TKIs targeting the VEGF/VEGFR axis and immunotherapies targeting PD-1/PD-L1 have also become the standard treatment for metastatic ccRCC, and these combinations are now recommended as the first-line treatment for metastatic ccRCC according to the latest European recommendations (48). However, there appear to be no biomarkers that can accurately assess the efficacy of immunotherapy and/or TKI therapy, as many biomarker-negative patients do not respond to these treatments. We innovatively found that patients with Musashi-2 overexpression had greater sensitivity to sorafenib, sunitinib, temsirolimus and gefitinib; conversely, patients with lower Musashi-2 expression were more sensitive to ICB immunotherapy, given the high expression levels of immunosuppressive factors in their tumors. These findings may help guide the development of better individualized clinical treatments for ccRCC patients.

Inevitably, our study also had some shortcomings. This study detected only the expression of Musashi-2 and PD-L1 in clinical specimens, and we should also explore changes in other immune checkpoints, metabolism-related genes and infiltrating immune cell subsets. The mechanism of Musashi-2-related metabolic reprogramming affecting immune infiltration of ccRCC needs to be further validated and explored *in vivo* and *in vitro*.

## Conclusion

We found for the first time that Musashi-2 expression was associated with immune infiltration in ccRCC, particularly showing positive correlations with CD4^+^ and CD8^+^ T cells and negative correlations with Treg cells and immune inhibitors. The Musashi-2 expression signature can be used as a novel indicator for predicting the clinical outcomes of ccRCC patients and will help us to deeply explore the roles of cellular metabolic reprogramming and immune infiltration in ccRCC.

## Data availability statement

The original contributions presented in the study are included in the article/**Supplementary Material**. Further inquiries can be directed to the corresponding authors.

## Ethics statement

The studies involving human participants were reviewed and approved by The Ethics Committee of Xiang’an Hospital of Xiamen University approved this study. The patients/participants provided their written informed consent to participate in this study.

## Author contributions

HL and HT conceived the project and designed the experiments. HL, XM performed the experiments. HL, XM, WZ, XY, WO, XP and RZ analyzed the data. HL and HT supervised this study. HL, XM wrote the manuscript and original draft. All authors contributed to the article and approved the submitted version.

## Funding

This study was supported by the National Natural Science Foundation of China (Grant No. 81972216).

## Acknowledgments

We appreciate all the participants for their efforts in the research process in this study.

## Conflict of interest

The authors declare that the research was conducted in the absence of any commercial or financial relationships that could be construed as a potential conflict of interest.

## Publisher’s note

All claims expressed in this article are solely those of the authors and do not necessarily represent those of their affiliated organizations, or those of the publisher, the editors and the reviewers. Any product that may be evaluated in this article, or claim that may be made by its manufacturer, is not guaranteed or endorsed by the publisher.

## Abbreviations

ccRCC, Clear cell renal cell carcinoma; VHL, von Hippel-Lindau; OS, Overall survival; PFS, Progression free survival; DSS, Disease specific survival; DFS, Disease free survival; RBPs, RNA-binding proteins; TMA, Tissue microarrays; IHC, Immunohistochemistry; TCGA, The Cancer Genome Atlas; TIDE, Tumor Immune Dysfunction and Exclusion; ICB, immune checkpoint blockade; TKI, Tyrosine kinase inhibitor.
