# Supplementary material for: Increased expression of the RNA-binding protein Musashi-2 is associated with immune infiltration and predicts better outcomes in ccRCC patients
Source: Front Oncol. 2022 Oct 21;12:949705. doi: 10.3389/fonc.2022.949705 (PMC9634258; doi:10.3389/fonc.2022.949705)
Supplement: Supplementary file 1 [file DataSheet_1.pdf]

Supplementary Materials for

Increased expression of the RNA-binding protein Musashi-2 is associated with immune infiltration and predicts better outcomes in ccRCC patients

Hui Li[1†*], Xiaole Meng[1,2,3†], Xuting You[1,3], Wenting Zhou[3], Wanxin Ouyang[3], Xin Pu[1,3], Runan Zhao[4], Huamei Tang[1,3*]

[1]Department of Pathology, Xiang'an Hospital of Xiamen University, Xiamen 361000, China

[2]National Institute for Data Science in Health and Medicine, Xiamen University, Xiamen 361000, China

[3]Organ Transplantation Institute of Xiamen University, Fujian Provincial Key Laboratory of Organ and Tissue Regeneration, School of Medicine, Xiamen University, Xiamen 361000, China

[4]Department of Pathology, Chang Hai Hospital, Navy Medical University, Shanghai 200433, China

†These authors contributed equally to this work.

Correspondence to:

Huamei Tang (tanghuamei2014@163.com) or Hui Li (lihui@xah.xmu.edu.cn)

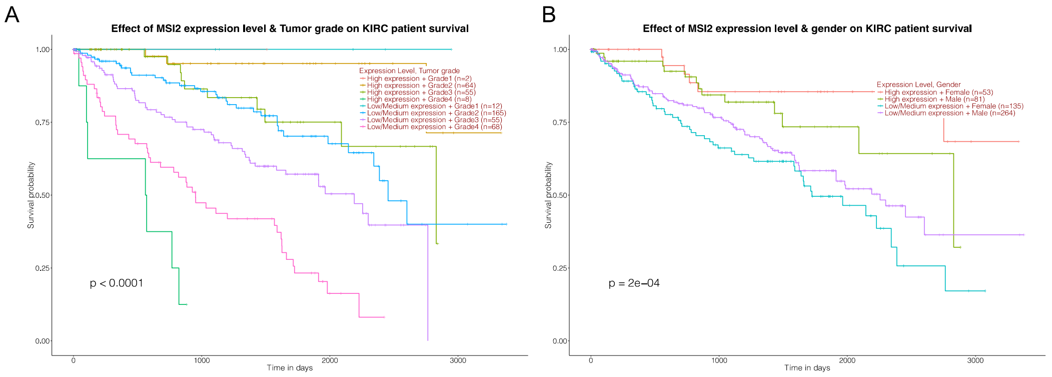

Fig.S1. Survival analysis of Musashi-2 with tumor grade and gender in 533 cases of ccRCC

A-B, The Overall survival (OS) of Musashi-2 with tumor grade (**A**) and gender (**B**) in ccRCC by using Kaplan Meier.

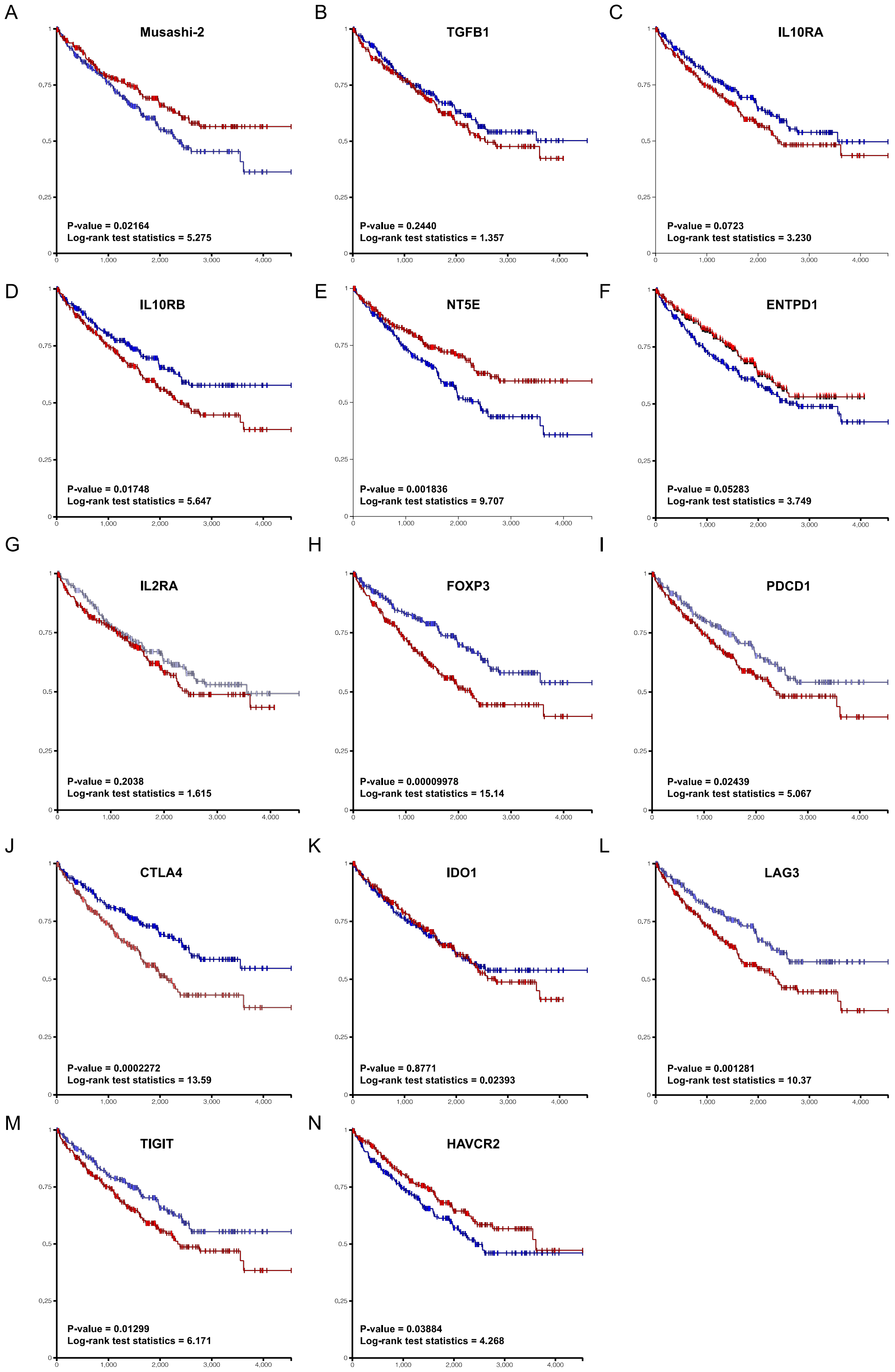

Fig.S2. Survival analysis of Musashi-2 and immune inhibitors in 602 cases of ccRCC

A-M, The Overall survival (OS) of Musashi-2 and immune inhibitors including TGFB1(**B**), IL10RA(**C**), IL10RB(**D**), NT5E(E), ENTPD1(F), IL2RA(**G**), FOXP3(**H**), PDCD1(**I**), CTLA4(**J**), IDO1(**K**), LAG3(**L**), TIGIT(**M**) and HAVCR2(**N**) in ccRCC by using Log rank test, the cutoff value was set at 50%, n=602.

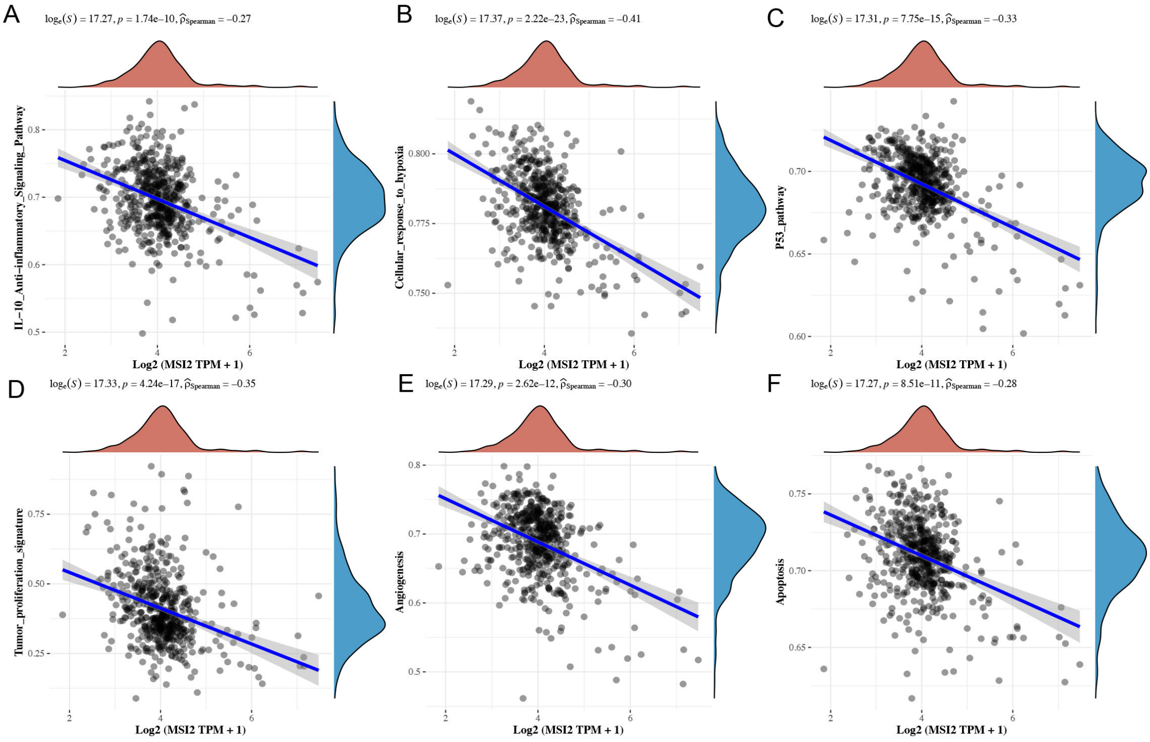

Fig.S3. Correlation analysis of Musashi-2 expression and signaling pathway score in 530 cases of ccRCC from TCGA

A-F, Spearman correlation analysis of Musashi-2 expression and IL-10 Anti-inflammatory signaling pathway (*p* =1.74e-10, cor =-0.27), Cellular response to hypoxia signaling pathway (*p* =2.22e-23, cor =-0.41), P53 signaling pathway (*p* =7.75e-15, cor =-0.33), Tumor proliferation signaling pathway (*p* =4.24e-17, cor =-0.35), Angiogenesis signaling pathway (*p* =2.62e-12, cor =-0.30) and Apoptosis signaling pathway (*p* =8.51e-11, cor =-0.28).

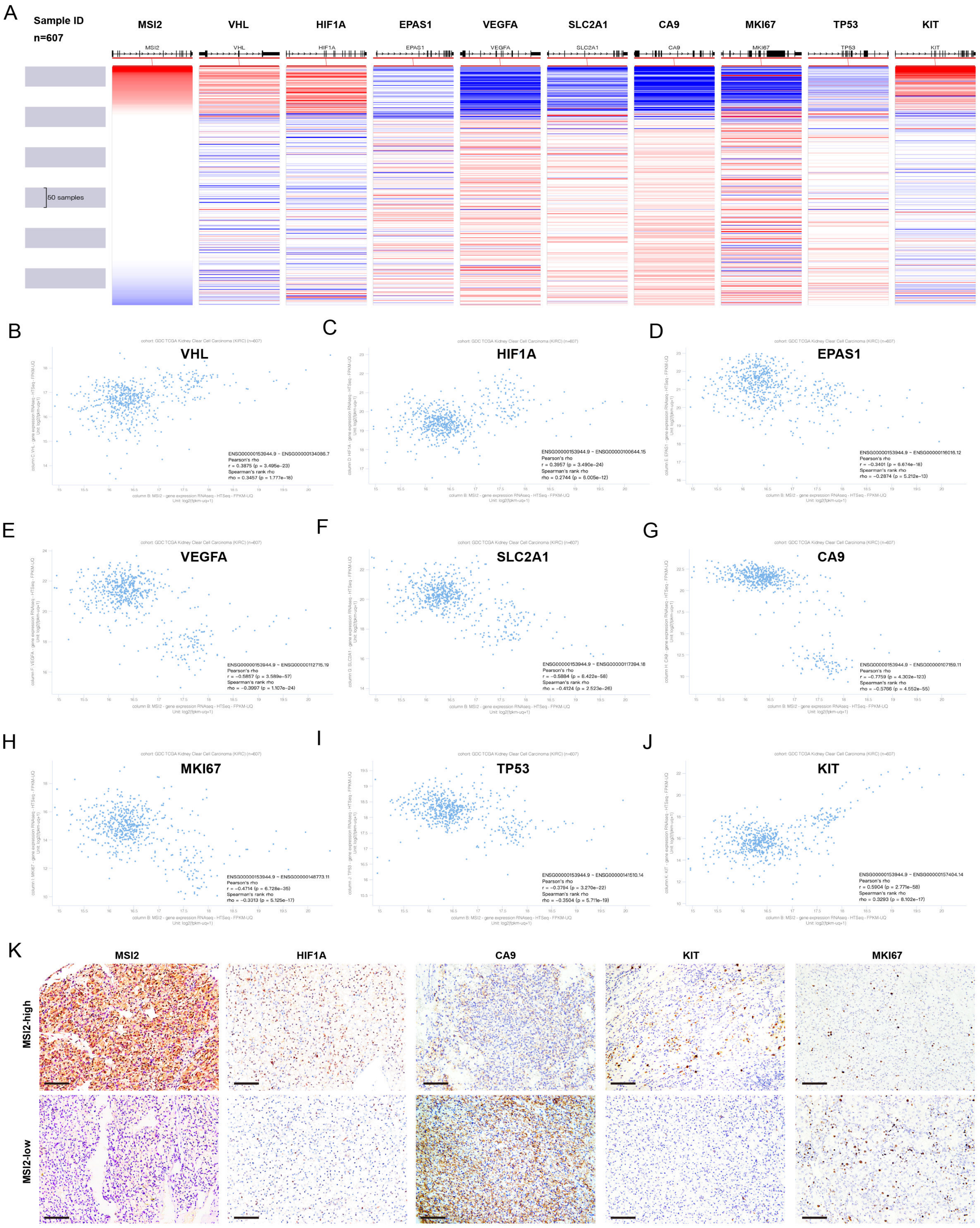

Fig.S4. Correlation analysis of Musashi-2 expression and metabolism-related genes expression

A, Heatmap of Musashi-2 and clinicopathological metabolic markers expression in ccRCC patients from GDC TCGA(n=607).

B-J, Associations between Musashi-2 and clinicopathological metabolic markers including VHL (*p* =3.495e-23, r =0.3875) (**B**), HIF1A (*p* =3.490e-24, r =0.3957) (**C**), EPAS1(*p* =6.674e-18, r =-0.3401) (**D**), VEGFA (*p* =3.589e-57, r =-0.5857) (**E**), SLC2A1 (*p* =8.422e-58, r =-0.5884) (**F**), CA9 (*p* =4.302e-123, r =-0.7759) (**G**), MKI67(*p* =6.728e-35, r =-0.4714) (**H**), TP53 (*p* =3.270e-22, r =-0.3794) (**I**) and KIT (*p* =2.771e-58, r =0.5904) (**J**) in ccRCC (n=607).

K, Representative image of immunohistochemical staining for HIF1A, CA9, KIT and MKI67 in Musashi-2 high and low ccRCC tissues. Scale bars, 100 μm.

Table.S1. Top 100 similar genes associated with Musashi-2 in ccRCC were detected by GEPIA2.

Table S1

Similar Genes

Gene Symbol	Gene ID	PCC	Gene Symbol	Gene ID	PCC	Gene Symbol	Gene ID	PCC
ATP6V1A	ENSG00000114573.9	0.9	ATP6V0A4	ENSG00000105929.15	0.78	PDHB	ENSG00000168291.12	0.76
TMEM213	ENSG00000214128.10	0.86	PCCB	ENSG00000114054.13	0.78	TBC1D24	ENSG00000162065.11	0.76
RALGPS1	ENSG00000136828.18	0.85	RP11-120K24.3	ENSG00000267868.1	0.78	TMPRSS2	ENSG00000184012.11	0.76
KIF13B	ENSG00000197892.12	0.84	TMEM33	ENSG00000109133.12	0.78	AREL1	ENSG00000119682.16	0.76
NNT	ENSG00000112992.16	0.84	RP11-728F11.4	ENSG00000254528.7	0.78	CDS1	ENSG00000163624.5	0.76
SLC16A7	ENSG00000118596.11	0.84	TMEM184C	ENSG00000164168.7	0.78	NUDT9	ENSG00000170502.12	0.76
PXK	ENSG00000168297.15	0.83	PEX7	ENSG00000112357.12	0.78	PIP4K2C	ENSG00000166908.17	0.76
LARS2	ENSG00000011376.9	0.83	SMIM5	ENSG00000204323.5	0.78	TBC1D4	ENSG00000136111.12	0.76
SACM1L	ENSG00000211456.10	0.83	ADAM1B	ENSG00000226469.1	0.78	COQ3	ENSG00000132423.10	0.76
TMEM116	ENSG00000198270.12	0.82	ATP6V1H	ENSG00000047249.16	0.78	ESRRG	ENSG00000196482.16	0.76
GABARAPL1	ENSG00000139112.10	0.82	TBC1D14	ENSG00000132405.18	0.78	WNK3	ENSG00000196632.10	0.76
FGF9	ENSG00000102678.6	0.82	HACD3	ENSG00000074696.12	0.78	ATP6AP2	ENSG00000182220.13	0.76
STRBP	ENSG00000165209.18	0.82	ATP5B	ENSG00000110955.8	0.77	C1orf168	ENSG00000187889.12	0.76
TRIM2	ENSG00000109654.14	0.81	SYNJ2BP	ENSG00000213463.4	0.77	EPB41L4B	ENSG00000095203.14	0.75
MCF2L-AS1	ENSG00000235280.2	0.81	MPPED2	ENSG00000066382.16	0.77	SH2D4A	ENSG00000104611.11	0.75
MOB1B	ENSG00000173542.8	0.81	UQCRFS1	ENSG00000169021.5	0.77	OXNAD1	ENSG00000154814.13	0.75
TFCP2L1	ENSG00000115112.7	0.81	IDH3A	ENSG00000166411.13	0.77	SLC4A9	ENSG00000113073.14	0.75
NR3C2	ENSG00000151623.14	0.81	AC013463.2	ENSG00000236283.3	0.77	GMPR	ENSG00000137198.9	0.75
AC019181.2	ENSG00000233255.1	0.8	RP11-368L12.1	ENSG00000260658.5	0.77	BMPR1B	ENSG00000138696.10	0.75
TMEM61	ENSG00000143001.4	0.8	FAM184B	ENSG00000047662.4	0.77	ATP6V0D2	ENSG00000147614.3	0.75
THSD7A	ENSG00000005108.15	0.8	SUSD1	ENSG00000106868.16	0.77	PFN2	ENSG00000070087.13	0.75
ATP5A1	ENSG00000152234.15	0.8	IQCH-AS1	ENSG00000259673.5	0.77	PDP2	ENSG00000172840.6	0.75
BSND	ENSG00000162399.6	0.8	SCNN1A	ENSG00000111319.12	0.77	TBC1D9	ENSG00000109436.7	0.75
CTDSPL	ENSG00000144677.14	0.8	RP1-206D15.6	ENSG00000213062.4	0.77	ZDHHC23	ENSG00000184307.13	0.75
PPARGC1A	ENSG00000109819.8	0.79	CMTM4	ENSG00000183723.12	0.77	PTPN3	ENSG00000070159.13	0.75
PLEKHB2	ENSG00000115762.16	0.79	EXOSC7	ENSG00000075914.12	0.77	GOT2	ENSG00000125166.12	0.75
ITPR2	ENSG00000123104.11	0.79	AP5M1	ENSG00000053770.11	0.77	NEDD4L	ENSG00000049759.16	0.75
TBC1D1	ENSG00000065882.15	0.79	C14orf159	ENSG00000133943.20	0.77	THRB-AS1	ENSG00000228791.7	0.75
UGT8	ENSG00000174607.10	0.79	SLC26A7	ENSG00000147606.8	0.77	GBAS	ENSG00000146729.9	0.75
SLC9A2	ENSG00000115616.2	0.79	ANGPTL1	ENSG00000116194.12	0.76	MAGI3	ENSG00000081026.18	0.75
CIPC	ENSG00000198894.7	0.79	RP1-80N2.2	ENSG00000226281.2	0.76	OGDHL	ENSG00000197444.9	0.74
THRB	ENSG00000151090.17	0.78	UQCRC1	ENSG00000010256.10	0.76	PFKM	ENSG00000152556.15	0.74
FREM1	ENSG00000164946.19	0.78	DLD	ENSG00000091140.12	0.76	DLAT	ENSG00000150768.15	0.74
						ALAS1	ENSG00000023330.14	0.74
